# Weaving spiritual care into nursing education: how the use of a self-assessment tool motivates students in a qualitative study

**DOI:** 10.1186/s12912-026-04514-1

**Published:** 2026-03-04

**Authors:** Joanne Lassche-Scheffer, Bart Cusveller, Tove Giske

**Affiliations:** 1 Centre for Diaconia and Professional Practice, VID Specialized University, Diakonveien 12-18, Oslo, 0370 Norway; 2 Faculty of Health Care, Viaa Christian University of Applied Sciences, Postbus 10030, Zwolle, GA 8000 The Netherlands; 3 Faculty of Health Sciences, VID Specialized University, Ulriksdal 10, Bergen, 5009 Norway

**Keywords:** Spiritual care, Nursing education, Competency, EPICC Tool, Nursing students, Motivation, Professional development, Qualitative research

## Abstract

**Background:**

Nurses have a critical role in providing spiritual care that improves the well-being of patients. Competencies for spiritual care are well described by the Enhancing Nurses’ and Midwives’ Competence in Providing Spiritual Care through Innovative Education and Compassionate Care (EPICC) network. Development of these competencies needs to be addressed by nursing education but how is yet to be explored.

**Objective:**

Explore how the implementation of the EPICC Spiritual Care Competency Self- Assessment Tool in a bachelor nursing program contributes to the development of spiritual care competencies in nursing students.

**Design:**

Qualitative study to explore experiences of nursing students regarding their development of spiritual care competency.

**Setting(s):**

Bachelor nursing education in the Netherlands.

**Participants:**

20 undergraduate nursing students.

**Methods:**

Individual interviews, Reflexive Thematic Analysis.

**Results:**

The students described developing spiritual care competency as a process influenced by student factors, the support from significant others, and the design and content of the curriculum. Students reported the use of the EPICC Tool as enhancing this learning process by providing reflection, support and knowledge about spiritual care. The EPICC Tool is mentioned as support to overcome barriers to start providing spiritual care. In this process, students opened up for spiritual care and recognised ‘the other’ as a human being. By building relationships they reported incentives to repeatedly provide spiritual care. By doing so, they built up competency and became intrinsically motivated for spiritual care. The mandatory use of the EPICC Tool is described as supportive.

**Conclusions:**

Developing spiritual care competency is a process influenced by many factors. This research shows that students experienced that the use of the mandatory EPICC Tool enhances this learning process. Educators should consider the use of mandatory assignments and threading spiritual care content throughout their curriculum to support the process of learning.

**Trial registration:**

clinical trial number: not applicable.

**Supplementary Information:**

The online version contains supplementary material available at 10.1186/s12912-026-04514-1.

## Introduction

### Background

Spirituality and spiritual care have become significant areas of research in health care and nursing [1, 2]. Nursing research has addressed spiritual needs and spiritual well-being in patients as part of nurses’ role in spiritual care [3–5]. Spiritual care is reported to improve quality of life, satisfaction, emotional health, physical functioning, and mental health of patients [4, 6].

Nurses need to develop competence for their role in spiritual care. Recently, researchers in Europe and beyond [7], have developed in the *Enhancing Nurses’ and Midwives’ Competence in Providing Spiritual Care through Innovative Education and Compassionate Care*, (or ‘EPICC’ network) an educational ‘EPICC Spiritual Care Education Standard’ (or ‘EPICC Standard’) for nursing competencies in spiritual care [1, 8, 9]. As spiritual care competence proves correlated positively to perceptions of spirituality [10] and the frequency in which spiritual care is provided [11], questions emerge as to how nursing students are best formed and educated to become competent in spiritual care.

Research [1, 2, 12, 13] confirms that integrating spiritual care in nursing education remains challenging. Research indicates it is essential for spiritual care learning to stimulate awareness of one’s own spirituality [2, 14, 15] making self-reflection an often-preferred learning strategy [16–19]. Furthermore, nurturing educational and clinical environments as well as personal student factors impact competency learning in spiritual care [8, 10, 14, 20]. Also, it is recommended in some literature that spiritual care education should be mandatory [2] and threaded through the curriculum [12, 16, 21, 22].

However, what we don’t know is how nursing students experience competency development in spiritual care in an educational environment where mandatory spiritual care content is integral to their curriculum [10, 13, 21, 22]. We don’t know much about what students think about mandatory learning strategies and how they help them to develop spiritual competency [23]. The objective of this study was to explore the experiences nursing students have with the mandatory use of a self-assessment tool in a bachelor nursing program and how it contributes to their development of spiritual care competency.

### The EPICC Spiritual Care Competency Self-Assessment Tool

For this study we used a validated self-assessment tool [24] that built upon the EPICC Standard, the so-called EPICC Spiritual Care Competency Self-Assessment Tool, (or ‘EPICC Tool’). To clarify the perspective from which this tool understands spirituality and spiritual care, we provide some background information. For spirituality, the definition was gleaned from the European Association of Palliative Care [25]:

‘Spirituality is the dynamic dimension of human life that relates to the way people (individual and community) experience, express and/or seek meaning, purpose and transcendence, and the way they connect to the moment, to self, to others, to nature, to the significant and/or to the sacred.

Spirituality and spiritual well-being are multidimensional, including:

1. Existential challenges (e.g., questions concerning identity, meaning, suffering and death, guilt and shame, reconciliation and forgiveness, freedom and responsibility, hope and despair, love and joy);
2. Value-based considerations and attitudes (e.g., what is most important for each person, such as relations to oneself, family, friends, work, aspects of nature, art and culture, ethics and morals, and life itself);
3. Religious considerations and foundations (e.g., faith, beliefs and practices, the relationship with God or the ultimate).’

For spiritual care the definition of the NHS Education for Scotland is used [26]:

‘Spiritual care is care which recognises and responds to the human spirit when faced with life-changing events (such as birth, trauma, ill health, loss) or sadness, and can include the need for meaning, for self-worth, to express oneself, for faith support, perhaps for rites or prayer or sacrament, or simply for a sensitive listener. Spiritual care begins with encouraging human contact in compassionate relationship and moves in whatever direction need requires.’ (p.7).

The EPICC Tool itself consists of four main competencies integrating knowledge, skills and attitudes, divided in 28 statements to be rated on a 5-point Likert scale [24]. The four competencies are:

1. Intrapersonal: awareness of the importance of spirituality on health and well-being and one’s own spirituality;
2. Interpersonal: engagement with the others’ spirituality and acknowledgment of the unique spiritual and cultural worldviews, beliefs and practices;
3. Assessment and planning: identification of spiritual needs and interventions;
4. Intervention and evaluation: providing spiritual care and evaluate its impact [27].

In addition, open questions are added to invite students to reflect on their strengths and weaknesses in spiritual care [24].

### Purpose and research question

The purpose of this study was to explore how nursing students experience the implementation of the EPICC Tool as an integral element of their bachelor nursing program and how it contributes to their development of spiritual care competencies. The research question was: What are nursing students’ experiences regarding their development of spiritual care competencies when using the mandatory EPICC Tool integrated in their curriculum?

## Methodology

### Research design and perspective

To explore students’ experiences with the EPICC Tool we used a qualitative approach with in-depth individual interviews to explore the subjective experiences [28, 29]. This interpretative research paradigm is practiced by many scholars in this field, studying the phenomenon of spirituality and lived experiences [22, 30]. The educational context is seen from the same perspective. As Biesta [31] asserts, education is an interactive process in which the interpretation of learning experiences plays an important role.

We used the approach of Reflexive Thematic Analysis [32–34] to guide our data interpretations. To make our perspective explicit [34], we began by reflecting on our positions as white researchers in the nursing field in Western European countries, with an interest in spirituality in nursing and education. We all have educational duties in addition to our research work. We used the Standards for Reporting Qualitative Research [35] to report this research which covers most areas of the extensive Reflexive Thematic Analysis Reporting Guidelines [36].

### Research setting

At Viaa, where data collection took place, a full-time (240 credit) program for a bachelor’s degree in nursing takes four years, divided in 4 modules of 10 weeks per year. In the program the topic of spirituality and spiritual care in nursing is consistently addressed in lectures, classroom meetings, small group discussions and individual assignments. The conceptual basis for the nursing program is the Neuman Systems Model [37], which explicitly addresses the patient’s spirituality as a dimension of nursing.

Throughout the program, a ‘Personal Professional Development (PPD) line’ (of 8 credits), facilitates students’ reflection on their professional competency development in general. This means, student learning is monitored and supported in individual meetings and group meetings of 12 students by the same tutor over the entire four years. As part of the PPD line, students process models and theories, for example through discussion and role play, to help them take responsibility for their own personal and professional growth. For students, doing assignments in the PPD line is required to earn credits.

At the start of the academic year 2023–2024, the EPICC Tool was implemented in the PPD line. As part of mandatory assignments and input for meetings with their tutor, students were required to fill out the EPICC Tool six times during the four-year program. In Table 1, an overview is given of spiritual care related learning activities in the content of the curriculum, in which the PPD line and the EPICC Tool are embedded.

**Table 1:** Overview of spiritual care content integrated through nursing curriculum of Viaa

Year	Learning activity	Content	Assignment	EPICC Tool
1	Lectures (6)	Spiritual care (3) and Neuman Systems Model (3)	Multiple choice test	
	Class meetings (2)	Neuman Systems Model and spiritual assessment	Case study, group discussion and oral presentation	
	Group meetings PPD* (2)	Recognizing meaningful situations Spirituality and the EPICC Tool	Written and oral reflection, group discussion and watching video clips. Filling in EPICC Tool	
	Individual meeting PPD	Competency development in spiritual care	Oral reflection on self-assessment EPICC Tool	1st time
	Internship	Spiritual care in nursing	Written reflection on spiritual care competency	
2	Class meeting	Social Relational Skills and existential questions	Exercises	
	Individual meeting PPD	Competency development in spiritual care	Oral reflection on self-assessment EPICC Tool	2nd time
	Lecture	Spirituality and ethics in illness, suffering and death	Multiple choice test	
	Internship	Spiritual care competency in clinical practice	Written reflection on spiritual care competency	
	Individual paper PPD	Competency development in spiritual care	Written reflection on self-assessment EPICC Tool	3rd time
3	Internship	Competency development in spiritual care	Written reflection on self-assessment EPICC Tool	4th time
	Individual paper PPD	Competency development in spiritual care	Written reflection on self-assessment EPICC Tool	5th time
4	Internship	Spiritual care competency in clinical practice	Written reflection on spiritual care competency	
	Individual meeting PPD	Competency development in spiritual care	Oral reflection on self-assessment EPICC Tool	6th time

*PPD is the abbreviation of the Personal Professional Development line in the curriculum

### Participants

At the beginning of the academic year 2024–2025, the number of nursing students in the research setting was 710. Students were recruited using purposive sampling, with the aim of capturing a broad range of experiences with spiritual care. The recruitment strategies employed included the use of volunteer sampling through open invitations to all students of all study years and all study routes, snowball sampling via participant networks, and targeted recruitment to achieve maximum variation [38, 39]. Analysis of interviews was already started during the recruitment of respondents. When it became apparent that students with an affinity for spirituality were more participating, we decided for analytic adequacy to actively invite students who struggled with spiritual care to have an interview. Four of them were included in the sample.

The recruitment resulted in 20 participants. Most of them were third- and fourth-year students and between 20 and 24 years old. In total, six male and fourteen female students were included. At that time, most of the students had already taken the EPICC Tool once or twice. Six of them had a prior vocational education as a nurse. A brief overview of student characteristics is shown in Table 2.

**Table 2:** Characteristics of the respondents

Year	Number
1	2
2	4
3	6
4	7
Other (delayed)	1
**Age**	**Number**
< 20	2
20–24	13
25–30	2
> 30	3
**Gender**	**Number**
Female	14
Male	6
**EPICC Tool used**	**Number**
0x	2
1x	6
2x	9
3x	3
**Pre -education**	
High school	10
Pre-University	4
Vocational study as a nurse	6

Decisions about sample size were guided by analytic adequacy and interpretative richness [34, 39, 40]. We used the concept of ‘information power’ [38, 39] to decide if our sample was appropriate for this study. For this study, the information power was guided by the relatively narrow focus of the study, the fact that the sample comprises exclusively members of the student population, the personal manner in which the data was gathered, and the existing knowledge in the field of nursing education research [39].

### Ethical considerations

Ethical approval was received from the executive board of the Medical Ethics Committee of Isala in Zwolle, The Netherlands (# 231004). By mail, permission from the university board was given to implement the EPICC Tool in the curriculum and conduct this study.

Participation in interviews was anonymous and voluntary. Students who were interested received information beforehand and completed an informed consent form prior to the interview. Students’ contact data were stored on a password protected storage drive separate from the transcribed interviews so the researchers could not relate data to individual respondents. The respondents did not consent to their interview transcripts being publicly shared due to confidentiality concerns.

The first author holds an appointment as a lecturer at Viaa and is also a member of a research group in spiritual care in nursing. To maintain independence from respondents as a researcher, she was exempted during the study from educational responsibilities related to students and courses involved in this study. The first author has four years of experience in qualitative research and is currently pursuing a doctoral study. The other authors are experienced researchers in nursing ethics and philosophy or spiritual care and both supervisors of the doctoral study. The second author has also educational duties at Viaa, while the third works at VID Specialized University in Norway.

### Data collection

Data collection took place with individual semi-structured in-depth interviews among undergraduate nursing students one year after integration of the EPICC Tool in the curriculum. An interview guide was developed for this study to explore and gain insight into how students develop spiritual care competencies with questions related to the research question. The interview guide (Additional File 1) included prompts like ‘What are your experiences in learning to provide spiritual care?’ and ‘What are your experiences in using the EPICC Tool?’ and ‘What suggestions do you have for the programme to help students learn competencies related to spiritual care?’ A pilot interview was conducted to see if the questions were appropriate to explore the experiences of students. After evaluation with the student and the supervisors concerning the transcript of this pilot interview, no changes were made. The interview guide was used as a tool for open exploration to conduct dynamic qualitative interviews, following the insights of the interviewee and researcher. The interviews were conducted between September 2024 and January 2025. They took between 20 and 54 min, with an average of 36 min. Eighteen interviews were taken face-to-face in a quiet space at the nursing school. Because of health issues, two students were interviewed online using Teams. All interviews were audio recorded with a digital voice recorder.

### Data analysis

The interviews were analysed according to the process of Reflexive Thematic Analysis (RTA) [33, 34]. The first step, ‘Familiarizing with the data’ [34] (p.42–49), was done by writing reflexive notes after each interview by the first author. Following this, the interviews were transcribed verbatim in Dutch and English with the assistance of Autotekst, a tool developed by the University of Oslo and available in a personal, protected online student environment created for the first author. Both transcripts were carefully reviewed by the first author, and in cases where a doubt about the translation arose, a dictionary was consulted. These two rounds of checks ensured that the first author became immersed in the data. She concluded with drawing diagrams of the main elements to apprehend the whole dataset. Backward translation was not employed due to the first author’s ability to refer to the original Dutch version in the event of translation issues. There were two reasons for translating. Firstly, it provided the third author with the opportunity to read along during the analysis process, enabling her to do independent analysis of the data and act as a sounding board. Secondly, the aim was to publish in English. The second step of the RTA, ‘Generating codes’, was done in an inductive way to represent the meaning of the participants. Open, semantic and latent coding [34] (p.53–58) took place as a systematic process throughout the whole dataset with the help of MaxQDA software and resulted in more than thousand codes. It was a rather organic process to organise them in a meaningful way [34] (p.54). This was achieved by organising them into codes related to ‘spiritual care’, ‘developing competency’, ‘curriculum’ and ‘EPICC Tool’. Data collection and analysis proceeded iteratively. For the third step, ‘Combining codes into themes’, drawings of conceptual patterns were used [34] (p.85–92) (see Additional File 2) as well as reflective research journals, which were shared with the supervisors. During steps one to three, the first author was a visiting PhD candidate at a nursing school in the US for several months. This experience enhanced her awareness of her cultural assumptions and perspective. In step four, ‘Review the themes’, both supervisors read the reflexive notes and randomly the interviews. Regular analytic discussions functioned as a form of reflexive sense-checking, supporting transparency and depth in the interpretive process rather than achieve coding consensus [34] (p.97–104). The first author gave oral presentations of preliminary interpretations to two research groups of experts in the field and on two international research conferences. This facilitated step five, ‘Determine significance of themes’, discussing the importance of the themes in relation with existing theories in the field [34] (p.57). Steps three to five were flexible and iterative [33], by rereading interviews and codes by the first author and ensuring no significant themes were overlooked. Lastly, step six, ‘Report of Findings’, was completed for publication, where main themes and subthemes supported by interview quotations became like ‘chapters in a broader story’ [40]. Quotes were selected as illustrative for this descriptive way to ‘show’ the data content [34] (p.132), aiming for a balanced distribution of the different students. Drafts of the article were discussed in several rounds with the other authors. An almost final draft was sent by email to the respondents, together with one of the drawings and the question how they experienced our interpretation of the interviews. Five students responded to this. They were positive about this interpretation, and no one had additional comments.

## Results

### Themes and subthemes

Two main themes reflect students shared ways of talking about and understanding their development in spiritual care competency. The first main theme is that students reported their development in spiritual care as a process with distinct stages. The second main theme is that they described several factors that influenced their learning process. Both main themes have subthemes, which will be described in the paragraphs below. An overview of the themes and subthemes with a short description is given in Table 3.

**Table 3:** Overview of themes and subthemes of experiences nursing students had with the mandatory use of the EPICC Tool in their bachelor nursing program on their development of spiritual care competencies

Main themes	Subthemes	Description
Learning spiritual care is a process	Entering nursing education	Students are different when they enter nursing education.
	Overcoming barriers	Students need to overcome barriers before they start developing themselves in spiritual care.
	Following education	Following education gives students the tools to develop in spiritual care.
	Opening up for spiritual care	Opening up is a starting point for developing spiritual care competency when students see the ‘other’ as a ‘human being’ with his/her own perspective.
	Developing competency in spiritual care	Students develop competency by providing spiritual care and receiving rewarding reinforcements.
Factors influencing the learning process	Personal abilities influence learning	Students are different in how they develop competency.
	Support by others	Students are supported by educators, supervisors, peers and patients in their learning process.
	Content and design of the curriculum	The content and design of the curriculum support the learning process, especially the EPICC Tool which is embedded in the curriculum.

We first report on students learning process in spiritual care and, second, report on the influences (on their learning process) of which the EPICC Tool was a part.

### Learning spiritual care is a process

The first main theme is that students experienced the development of competency in spiritual care as a process with distinct stages as illustrated by this second-year student *‘I think it’s important that you consider it [spiritual care] as caregiver. What is important for the patient? But that it is difficult to learn, yes. … A process’* (R19, 231-232;236)^1^. It is a lived journey, beginning from each student’s own individual starting point when entering the nursing program, overcoming personal and professional barriers during the program, and reaching a professional awareness of spiritual care following education. This opening up for spiritual care is the recognition of patients as fellow human beings in need, which prompts students to provide spiritual care and, by doing so, to become more competent.

#### Entering nursing education

First, students’ experiences were different because of their personal backgrounds and approach to education. Some students mentioned they lacked the knowledge and social and relational skills required to provide spiritual care. Others had already experienced spiritual care from prior vocational training as a nurse, even if they were not consciously aware of what spiritual care was at that time. Students’ awareness of and attitude towards spirituality also differed because of their upbringing and earlier life events. Like a first-year student mentioned: ‘*I’ve been depressed several times … So, that makes spirituality very important to me’* (R16, 85) while another second-year student said *‘No, it [spiritual care] didn’t mean anything to me at first’* (R20, 27). Lastly, students differ in their ability to reflect on their own development, and their learning strategies range from avoiding spiritual care *‘I closed my eyes a bit when it was my turn’* (R19, 97) to learning consciously through trial and error, like this third-year student *‘I wasn’t really afraid to say something wrong. I said wrong things, but I learned from it’* (R3, 24).

#### Overcoming barriers

Second, learning spiritual care was not merely experienced as a matter of maturation, as students faced various barriers along the way. One is their ‘apprehension’ for the concept of spirituality, like this fourth-year student told ‘*When I use the term, I really think, well, I’m a bit scared, because I think, how am I going to explore this?* (R1, 30). Students experienced the concept as ‘big’ and ‘heavy’, as this fourth-year student described: *‘I think this is a bit too big. Yes, that makes me feel like a snot-nosed kid for raising it for discussion’* (R12,149). They felt it was about questions of life and death and difficult emotions, like this second-year student who told about a resident whose sister had just passed away *‘I found it difficult… I didn’t know how to approach it … yes, and then she doesn’t want to talk about it and then she gets angry again and thinks “Stop it”.’* (R20, 284; 286). Most students saw themselves as too young to deal with such situations early in their studies. A first-year student mentioned: *‘How do you ask questions? Sometimes they are quite sensitive questions. When you are quite young, you find that very difficult’* (R14, 168). Fear of being incompetent was another barrier. Students initially struggled to find the right professional attitude towards spiritual care due to the sensitive subject. They found it difficult to decide how much presence or distance is appropriate for a nurse when providing spiritual care. A fourth-year student mentioned: *‘And what is your professional attitude … and how do you do that? Because you can’t cry with someone, because that’s not professional’* (R5, 202). Another challenge they faced was to find balance between their own vulnerability when faced with difficult situations. A third-year student, who described her experiences with an internship in Ghana: *‘Giving spiritual care or being there for someone, but I just didn’t dare to do it yet. Because I was afraid, I just didn’t feel comfortable. I really thought, what are they going to think of me and stuff like that.’* (R15, 676–680). Students found it difficult to take care of themselves. The confrontation with their own history or life circumstances in contact with patients was a barrier for them. Some of them first needed to learn to have self-care before they could provide spiritual care to others. This fourth-year student shared about her internship in a hospital: *‘They always say, you must take care of yourself first, and only then you can take care of someone else … I noticed that, when I was diagnosed that I had a tumour … I found it extremely difficult to go to work … At work I only say people who might have cancer… Then I said to the manager for myself, well, I just can’t do it now’* (R7, 71;76–77). Another barrier was the struggle how much time to spend on spiritual care, as this fourth-year student illustrates: *‘There are so many more people who need to have a conversation. And you know, it doesn’t always fit. But I think, look at it. … I think people are so happy when you sit down with her for five minutes … Then the whole day is good again. And then sometimes they say, we don’t have time. And then you think, well, those five minutes.*’ (R1, 201).

#### Following education

Third, by proceeding through the curriculum, students experienced that they were given tools to gain competency in spiritual care and to overcome barriers to provide it. They were taught what they need to know about spiritual care in lectures and training in social and relational skills. A second-year student explained this as follows: ‘*That you indeed in the first year get theory about spiritual care. And also indeed that assessment instruments in the first year … And then you start to deal with it more consciously. And to take it with you during the training, in the competence tests for example’* (R6,199). Exposure to spiritual care and exercises to develop skills and attitude were facilitated in the classroom with peers, as well as in their private circle with elderly or frail relatives. During education, internships or ‘clinicals’ provided opportunities in real-life situations to try their hand at what they had learned, which contributed greatly to their understanding of spiritual care. As expressed by this third-year student: *‘I think it was also in small group classes, when we were going to talk about patient cases, … and the spiritual part that was part of it. … Those techniques at Social-Relational Skills-lessons. … But really, I think it’s even nicer to apply spiritual care in real situations, in internships’* (R8, 80–81; 211). The programme, lastly, provided reflection assignments, through which students learned to consider their personal and professional attitudes after conversations with patients. A first-year student said: *‘But it [spiritual care] also was a topic in the Personal Professional Development-meeting I had last week. I was hardly aware of a piece of spiritual care that I do. Because I do a lot automatically, without really thinking about it’* (R14,12).

#### Opening up for spiritual care

Fourth, at some point students recognized they have their own perspective on things that happen in patient care. At the same time, they realised that the patient is a human being with his or her own history, family, life and dreams for the future that may or may not be like their own. Thus, students reached a stage where they could open up towards that perspective. They started to ponder questions such as ‘What if I were that person?’, or ‘What if it was one of my own family members?’ as this fourth-year student described ‘*It’s not just a patient. I think that’s also something you can see over time. That you think, this is a patient and they are apart from me. And that’s very good, of course. But that you also think, there’s a lot more behind it. He also has a family*’ (R11, 358). They started to see the other as a human being, like a fourth-year student mentioned: *‘In the end, you are all human and they are also vulnerable to you’* (R7, 102). This realisation helped them to initiate conversations and build relationships with the patient as a first-year student illustrated: *‘To be honest, I always try to put my profession aside a little when I’m providing care. It’s just two people together, enjoying each other’s company, and I use my skills to perform a few tasks and then we have a really nice conversation’* (R9, 37–38). This turning point occurred mostly between their 2nd and 4th year. Some students reached this stage early in their education, due to their own openness to and interest in others. Others needed more time, because they never encountered different perspectives or were not interested. An example is a second-year student, who was sceptical to be ‘forced’ to spiritual care when at first being indifferent to it, but argued for situations in internship to have a conversation with a patient and to be asked afterwards by their supervisor: *‘Well, is it different from how you do it? … what you think about it? That such a person looks at life in that way, different from how you would look at it?’* (R19, 161). Primarily during their internships, they encountered situations that made them start to think about it. At school, they practiced to some extent, but in clinical practice they encountered real-life situations where they felt helpless. These uncomfortable moments made them realise that they didn’t know what to do. They also realised during their second internship, when they were less occupied with the technical side of nursing that they might be the only person the patient saw in a day or week. The same student told later: *‘I find it difficult to take on spiritual care. Especially, in my first internship, yes, … But in my second internship, I investigated it a little more. Why did this patient want this? … Let me tell you, it’s beautiful! … then you get curious, and you must look at why they think so’* (R19, 12–13;19; 59).

This, they feel, was the starting point of providing spiritual care. This triggered their development of spiritual care. A fourth-year student elaborated on this: *‘So, just start that conversation and try to be open for it. Because once you’ve done it, you learn from it again’* (R10, 286–289).

#### Developing competency in spiritual care

Fifth, developing competency in spiritual care is the last stage in the learning process of providing spiritual care. As a result, the more students practice spiritual care, even by trial and error, the more they learn how to practice it. A third-year student told: *‘As I said earlier, I think it’s something you can only learn in practice. … The spirituality is so different, so unique and so vague. You cannot learn it from a book. … I really think that this is such a do-do-do subject’* (R3, 38–39; 340). When students provided spiritual care, they experienced rewarding reinforcements, stimulating the learning process and motivating to continue providing spiritual care. Another third-year student gave advice to other students: *‘To just sit quietly with someone, with a client. And just experience what can come out of it. And that also motivates you to go further and to get further into it. So, yes, that. Yes, I am enthusiastic in any case (laughs)’* (R4, 274–275). These reinforcements came in the form of gratifying moments and deep conversations with patients. Students were known by name by patients and got positive feedback from them as this fourth-year student who worked on an oncology ward illustrates: *‘So you see how important it is that you can get such a bond in such a short time with people. And that they still recognized me after three months. And that they immediately knew who I was’* (R7, 87). They also experienced job satisfaction when they see their patients happy or hear from family members that their loved ones looked happy. Students even experienced health benefits themselves, such as lower mental stress and frustration after a day’s work, and increased motivation and energy. A second-year student mentioned: *‘I think, for example, a meaningful day or that you feel mentally healthy. And physically, I think, too. … Yes, that residents just … become happier, that they are happy. Or that they thank you if you have done something for them’* (R6, 76;80) which was approved by a third-year student *‘I have seen … in the spiritual care in the small things, which can make the biggest difference … that I think it is less(thinks)… mentally stressful, I think. … I think it is more fulfilling that way instead of being very stressful’* (R13, 47–48). This experience seems to create a self-reinforcing cycle that makes students more motivated as well as more competent to provide spiritual care. This fourth-year student expressed: *‘I noticed that it does have an influence when you put more time and effort into someone. That was a kind of encouragement, and I thought it is easy. I mean, it is not always easy, but it is useful’* (R11, 237).

### Factors influencing the learning process

The second main theme is that students described that their process of developing competencies in spiritual care is influenced by several factors. A fourth-year student summarized them: *‘I do have my own view on what I find important in terms of spirituality. Because I have developed that. I think that’s partly… Yes, it’s also because attention is paid to it at school. And also because you see in practice that it doesn’t have to be that big. … So, I think it’s a combination of both, my own learning process and also the supervisors around me’* (R17, 55–61). These factors can be divided into the students themselves, to the support they receive from significant others, and to the content and the design of the curriculum in which, lastly, the EPICC Tool is embedded.

#### Personal abilities influence learning

First, students bring their own unique backgrounds which influenced their ability to develop spiritual care competency. Students mentioned how their backgrounds in prior education, work experience and life events influenced their ability to learn and develop, like this fourth-year student said: *‘I do think that you can open up more easily and that you have easier casuistic if you have experienced more, if you have a backpack, that you can reflect more on yourself’* (R7, 216). This is also related to the awareness of and attitude to spiritual care, like this second-year student mentioned: *‘It [spiritual care] didn’t interest me at all …You live in your own world, you are quite in a bubble’* (R19, 97;134). Accordingly, as another fourth-year student said, there are differences in doing assignments and learning strategies related to spirituality: *‘It also differs what kind of student you have in front of you, because one thinks it’s more important than the other’* (R10, 229).

#### Support by others

Second, supervision from teachers at the university and supervisors in clinical practice supported student learning. Students reported it as important that teachers and supervisors explain and model how to provide spiritual care. As this fourth-year student told: *‘If teachers had personal stories from the work field, I thought that was very engaging, but I also learned a lot from that’* (R2, 201). The same applied to peers in the classroom when students shared examples of spiritual care in case studies and reflection meetings after internships. An almost graduated student who worked in home care experienced: *‘You talk to each other at school, under the guidance of teachers, but also because you hear experiences from other people in other working conditions or other fields of work. We were in a very mixed class with people from psychiatry, people from nursing homes, from hospitals’* (R4, 40). Unwillingly, supervisors sometimes stimulated students by providing contrasting input, when spirituality was not addressed or just mentioned in very general terms. A third-year student told the spiritual care intervention for residents with dementia was not appreciated by the supervisors: *‘I like to use humour and stuff, but some supervisors don’t think that appropriate at all. … But that’s just my way of giving it a bit of spirit … just making a connection’* (R13,121–122). Additionally, feedback from patients influenced the way students provided spiritual care. This is illustrated by a fourth-year student working with a patient with aphasia: *‘I took the time for her … I let her make contact… and I noticed she was much easier to work with … she was much happier, and she thanked me a lot …That was a kind of encouragement’* (R11, 235–237).

#### Content and design of the curriculum

Third, theory about spiritual care helped students to understand the concept of spiritual care, how layered it is, and how to apply a holistic approach to practice. A second-year student reported: *‘What it actually meant, spiritual care. In what ways is it seen. That it can be on all levels. On a social level, on a psychological level, on a religious level. Actually, it can also be done in different fields’* (R6, 31). Specifically, students mentioned the Neuman Systems Model, a nursing theory with a holistic approach to patients’ wellbeing, as well as the EPICC Tool and other tools to assess spiritual care, like this third-year student *‘From the first years we have received a lot of NSM [Neuman Systems Model]’* (R3, 8). Third- and fourth-year students especially appreciated how spiritual care is integrated in many of the classes and assignments, as they felt focus on spirituality helped them to learn its importance for nursing care. A first-year student expressed this: *‘At school it [spiritual care] is valued quite highly, so it is something that returns in things a lot. I too think that is important, that it deserves a lot of attention, that it is not something that is relevant now and then over a very long time, or maybe only once, because then it just disappears, and other things come in its place’* (R14, 268).

The integration of theory and practice as well as working in small groups, furthermore, facilitated the development of spiritual care competencies. To learn spiritual care, students highlighted various opportunities for practical experiences through internships. Internships in different areas of health care helped them to apply spiritual care in real-life situations and to see that simply ‘doing’ it is necessary for competency. This fourth-year student illustrated: *‘But above all, I think that it [spiritual care] has come back most during the internships. Because it is the competence [about spiritual care] in the rubric that you really must pay attention to. And you also must prove that you have done it. So, then you think more about it. So, I think that helped the most’* (R5, 25–27). In addition, the design of the PPD line was helpful as it integrated lessons in spiritual care and reflections. It made students reflect on their personal and professional development in meetings with their tutor, providing inroads for students to become more aware of spiritual care competence. Another fourth-year student told: *‘I did discuss it [spirituality] with my PPD teacher a few times. … For myself, I didn’t know it could be so accessible. … That was also part of my learning process’* (R15, 271; 278; 282). Lastly, when students did mandatory assignments related to spiritual care during internships, it helped them to focus. It is notable that the mandatory assignments were acknowledged as helpful and necessary in the students’ learning process. Students expressed that these mandatory activities helped them to start thinking about spirituality, recognise its value and develop their intrinsic motivation to provide it. Thus, students found the recurrent use of a framework for self-reflection helpful. A third-year student reflected on this: *‘You just consciously dwell on it again like, oh, I can still learn something here and this is something I can work on in the coming year’* (R8, 524–525).

#### Use of the EPICC tool

Fourth, students described the use of the EPICC Tool, in particular, as an important support on their own learning process and on the content and design of the nursing program.

Students expressed that the EPICC Tool facilitated them to understand what spiritual care is in terms of knowledge, skills and attitudes. The four EPICC competencies provided content to it. A fourth-year student talked enthusiastically about it; ‘*Yes, I really liked how you, from intra-personal to inter-personal, if I say it correctly, with the competencies … Yes, first also really looking at yourself and also with knowledge, skills and then the attitude that is in it. That you really look at it in different ways, about being open, being respectful to each other, values, convictions’* (R7, 71). The language of the EPICC Tool provided students with insight into the concepts of spiritual care and spirituality as this fourth-year student mentioned: *‘Spirituality is a bit vague, a bit abstract. But it [the EPICC Tool] gives some concrete direction of what it is about*’ (R10,168). In the PPD line, filling out the EPICC Tool is combined with reflection on their self-assessment scores. Reflection helped students to become aware of spiritual care and evaluate their attitude towards it. A first-year student described the experience with the EPICC Tool as: *‘Yes, okay, it [the EPICC Tool] always makes you think. Well, your own actions and you know, how do I give content to spirituality, and it is always good for your self-reflection, yes’* (R16, 23). Students reported that the EPICC Tool brings spirituality from the unconscious to the conscious level. The structure of the EPICC Tool helped them to see that spirituality can manifest itself at different levels of competence and in different areas of expertise, like this second-year student expressed: *‘I think that you can indeed, on more levels, give spiritual care, well, how should I put it? That spirituality can be expressed in different areas’* (R6, 113). It helped them to shape their learning process by identifying specific areas for improvement. Two students reflected on taking the EPICC Tool: *‘I think it’s a really nice tool to use, because you can also see how you yourself are growing* (R18, 249); *‘Then you realize that a lot has already changed. … It is a manner of growth that you also experience yourself’* (R4, 168;189). Thus, the EPICC Tool was valued for its aid in monitoring and supporting personal and professional growth in spiritual care throughout the educational programme, like this fourth-year student told: *‘I think it’s good if you fill it in every year, so you can see the growth or where you might have held back a bit’* (R11,134). Another fourth-year student experienced the EPICC Tool as a reflection tool, that might support inner motivation to become a nurse, while admitting that in the first years of education that motivation was mainly external: *‘I think that in itself … is convenient … and especially for the first-year student, because you first must learn how to reflect. In the beginning you just write something down and then you think, well, let’s see if I can get away with it. Yes, that’s how it works (laughs). … And what did I learn? … I think that happened when I was doing the minor. … Then you know thìs is what I want! So, you really take your education for yourself. … I think that at some point in your third year is also a bit of a turning point’* (R10, 137–138; 145–146).

## Discussion

We have reported in a ‘descriptive way’ on the experiences nursing students had with the mandatory use of the EPICC Tool in their bachelor nursing program and how it contributes to their development of spiritual care competency. We will discuss our interpretation of the significance of our findings [34] (p.132) both in relation to research and theories.

### Significance for education

We discuss three points that have significance for education.

Firstly, that ‘opening up for spiritual care’ is a ‘starting point’ for developing in spiritual care competency. Students consider the discovery of ‘the other’ as a ‘human being’ with his or her own perspective, as a ‘person’ to be fundamental to the learning process of spiritual care. This is in line with Giske and Cone [41] and Haugland and Giskes’ [42] findings that opening up and being able to move the attention from self to the person you care for is essential for learning spiritual care. They described the process of discovering patients as a person as ‘enlivening the person’ [42], which is visible in some reflections of students. Seeing the other as a human being doesn’t happen automatically; it seems to be a journey of ‘discovery’. As this ‘discovery’ is fundamental for students, educators should facilitate this process. According to Whelan [15], this can be facilitated by educators when they give space in their curriculum for knowledge about multiple worldviews, for developing open and respectful attitudes and educate interaction skills. Then, ‘Such ways of knowing help students to think about patients’ humanity’ [15] (p.169).

Secondly, our findings showed that when students started to provide spiritual care, they experienced ’rewarding reinforcements’ that motivated them to continue to provide spiritual care. We interpreted this as intrinsic motivation for spiritual care. This resonates in what Alshakhshir et al. [21] call ‘consequences’ as the enhanced nurses’ spiritual well-being and satisfaction and what Ryan and Deci [43] found where people developed ‘pure interest and curiosity’ as elements of intrinsic motivation. In motivation theories this is called the internalisation and integration of what students consider important [43, 44]. As personal values and a belief that spirituality is part of nursing becomes integrated in students, they expressed the further development of spiritual care competency as a challenge which they enjoyed [43–45]. Haugland, Lassen & Giske [46] describe this process as the professional formation or maturation of nursing students.

Thirdly, if intrinsic motivation is such a ‘trigger’ in learning, how can we enhance students’ motivation? In contrast with the study of Alshakhskir [21] the majority of our students did not express the ‘willingness’ or interest to learn spiritual care, due to different barriers. The challenges in helping students to ‘overcoming barriers’ in learning spiritual care are addressed in many studies [2, 11, 13, 47]. These studies advice nursing education to weave spiritual care content and assignments through the program and to include it as part of exams. This is done in our study at Viaa. By ‘following education’, students had to carry out many mandatory assignments. Although students did not always like them, they seemed to stimulate awareness of spirituality by reflection and helping students to overcome barriers in starting to provide spiritual care. While some authors suggest voluntary participation in education is positively linked with achieving competence [21, 22], our study suggests that students, even if they expressed resistance to spiritual care, appreciated mandatory participation. They needed a structure with mandatory assignments before they reached a turning point in motivation, which occurred mostly between the 2nd and 4th year of their study. Evidence suggests mandatory assignments in spiritual care can move students from external to internal motivation [2, 46]. Haugland and Giske [42] describe how ‘musting’, meaning that students must take part in mandatory assignments, facilitated students to overcome their fears and barriers. This also aligns with Ryan and Deci [45]. Their self-determination theory is useful for understanding the factors that facilitate or undermine intrinsic motivation and is relevant in educational settings. It emphasises that when people’s basic needs for relatedness, competence and autonomy are met, they can be encouraged to transition from no motivation to intrinsic motivation [43, 44]. In our study, students reported that they became aware of their level of competency and felt autonomy in shaping and monitoring their learning process by using the EPICC Tool regularly through their education.

### Methodological considerations

In line with its interpretative design, the situatedness of the study has been described [36]. It was carried out in a particular country at a particular university which has its limitations.

Firstly, there is the risk of social desirability bias [48] as students were interviewed in their own institutional context by an interviewer employed at the same institution. We used strategies to identify and reduce that risk [48]. Strategies included the planning of the interviews, and the exemption of interviewer from student assessment. Also, the interviews were held in a quiet room and began with neutral background questions to ensure a relaxed atmosphere [48]. The interviewer did build rapport, as became clear when evaluating each interview. It was also an advantage that the interviewer was an insider, so that interviews were not interrupted by clarification questions about the curriculum. Afterwards, students reported that they felt listened to and that it was nice to talk about their experiences. As interviews were held on a voluntary basis, there could have been a self-selection bias [38] which could have an possible impact on generalisability. We used several strategies to recruit a maximum variation of experiences to enhance the ‘information power’ [39] of our study and reduce the risk of self-selection bias. As analysis progressed, later interviews primarily deepened, nuanced, and sometimes challenged the developing interpretations rather than introducing entirely different analytic directions. We interpreted this that our sample had enough information power for this study.

Secondly, spirituality and spiritual care is always place-, culture- and time-specific [15, 22]. Therefore, we have been transparent regarding the program we recruited students from, and that we worked to recruit students with diverse experiences related to spiritual care education. As the interviews were conducted in Dutch and translated to English, and the analysis was carried out in English, we have been transparent about the processes we have used to safeguard that meaning has been maintained. We have also consulted with respondents regarding our interpretation. We have presented our findings several times in international contexts to check its coherence and appeal.

Thirdly, consideration related to transferability of results to other nursing programs with different curricula and different cultures. The impact of spiritual care education is shaped by cultural and religious contexts [16]. For this study we used Reflexive Thematic Analysis [33, 34] to make our framework of interpretation explicit to enhance transferability [36]. The most prolific authors in the field of spiritual care education in nursing belong to the EPICC network and are primarily based in European health care and educational context with primarily Christian values [49, 50]. The EPICC Tool was developed with participants from outside Europe, namely Ghana and the US. The EPICC Tool has been translated into more languages, and in the future it will be interesting to keep track of potential impact of integrating the EPICC Tool in nursing programs worldwide [51, 52] as well as in longitudinal studies [13, 16].

## Conclusions

This study, done in one cultural setting at one institution, describes experiences of nursing students, who reported developing spiritual care competency as a process influenced by many factors. The factors are related to individual students, the support from significant others and the curriculum. Within students’ accounts, certain features of the use of the EPICC Tool enhances this learning process by providing reflection, support and knowledge. It seems to help students to overcome barriers to start to try out to provide spiritual care. When students reach the stage where they recognise ‘the other’ as a human being, students start to internalise and integrate spiritual care provision and become intrinsically motivated to develop their spiritual care competencies. Mandatory assignments addressing spiritual care seem to activate this process. So, this study is an illustration of what can happen with the motivation of nursing students for learning spiritual care. We suggest using the mandatory EPICC Tool as an integrated part of the curriculum to enhance motivation for developing spiritual care competence. Educators could consider how to apply this in their own situation, using this study as an example.

## Supplementary Information

Below is the link to the electronic supplementary material.

Supplementary Material 1

Supplementary Material 2

## Data Availability

Data are not publicly available due to confidentiality and consent restrictions.

## Footnotes

1 This reference (R19, 231–232; 236) means Respondent Number 19, quote is lines 231, 232 and 236 from transcribed interview.

## Abbreviations

EPICC: Enhancing Nurses’ and Midwives’ Competence in Providing Spiritual Care through Innovative Education and Compassionate Care, a global network of nursing/midwifery educators and practitioners and allied health professionals.
EPICC Standard: EPICC Spiritual Care Education Standard, a framework for 4 competencies in spiritual care divided in knowledge, skills and attitudes.
EPICC Tool: EPICC Spiritual Care Competency Self-Assessment Tool, a self-assessment tool for nursing students based in four spiritual care competencies.
PPD: Personal Professional Development line in the nursing education program which facilitates students’ reflection on their professional competency development.
RTA: Reflexive Thematic Analysis, a way of analysing qualitative data according to Braun & Clarke
Viaa: Viaa Christian University of Applied Sciences, Zwolle, the Netherlands
