# Supplementary material for: Weaving spiritual care into nursing education: how the use of a self-assessment tool motivates students in a qualitative study
Source: BMC Nurs. 2026 Mar 4;25:346. doi: 10.1186/s12912-026-04514-1 (PMC13069700; doi:10.1186/s12912-026-04514-1)
Supplement: Supplementary file 1 — Supplementary Material 1 [file 12912_2026_4514_MOESM1_ESM.docx]

## **Additional file 1. Interview guide in-depth interview**

## Interview guide in-depth interview on experiences with EPICC Tool

Interviews will be conducted with nursing students during academic year 2024/2025, when they have used the EPICC Tool at least twice for an education assignment.

### What needs to be arranged in advance

- Invitation to interview with information letter and consent form.
- Small room within Viaa to talk quietly.
- Recording equipment for sound recording (batteries).
- Printed consent form
- Printed questionnaire with background information.
- Little attention after interview.

### Interview guide

| Introduction | |
| --- | --- |
| *Introduce the conversation with the following information and questions in this order* | |
| **Intro** | My name is Joanne Lassche and I am a researcher of the research group Spiritual Care in Healthcare. I am also a lecturer at the Nursing programme.  I am very happy you want to participate in this interview and research. Is it true that you received the information about this research? Do you have any questions about this? |
| **Subject of research** | This interview is about what you will share about your learning process of spiritual care. |
| **Time duration** | The interview will take about 45 minutes. |
| **Stopping early** | If you decide during the interview that you do not want to continue, you can and may say so. We will then stop the interview. |
| **Recording** | As you can see, I have brought recording equipment, which will be used to record this interview. I need this, so that I can transcribe the interview later. I need your permission to do this.  **Will you sign the consent form?** |
| **Background information** | Before I start the recording, I would like to ask you to answer a few questions. I need the answers to make an assessment about the representativeness of my interview data. The questions are similar to those you encountered in the EPICC Tool.  **Would you like to complete these questions now?** |

| Consent statement research into experiences with the EPICC Self-assessment Instrument 'Spirituality in Nursing' in education |
| --- |
| **Give consent: participate in research**   - I have read the information letter and know what this research is for and what I will be doing. - I know what research data will be collected and how it will be stored. - I choose to participate in this research. I know that I can stop the study at any time. I also know what happens to the research data if I stop. |
| **Give permission: processing personal data**   - I consent to the researcher collecting, processing and securely storing my personal data for this study. - I consent to audio recording for this research. |
| **Name of participant:**  **Date:**  **Signature:**  **Name of researcher: Joanne Lassche-Scheffer**  **Date:**  **Signature:** |

| Questionnaire general background information |
| --- |
| This data will be stored in a different place from the typed-out interviews and the signature of your consent form, so personal information cannot be linked to the outcomes. |
| **Will you tell us something about your education and how you came into contact with the EPICC Tool by answering the questions below?** |
| 1. Which study component are you currently working on?   o Module IZ  o Module AZ  o Module PZ  o Internship FN2  o Internship FN3  o Internship FN4  o Minor  o Advisory project  o Other, namely |
| 1. What year of study are you in?    - Year 1    - Year 2    - Year 3    - Year 4    - Other, namely |
| 1. Which educational route are you following?    - VDV    - VVV    - VVK    - VVN    - Other, namely |
| 1. For which assignment did you complete the EPICC Tool (multiple answers possible)?   In preparation for...   - - PPD Discussion LZ   - PPD Discussion IZ   - PPD Reflection FN2   - Clinical assignment FN3   - PPD Reflection FN3   - PPD Discussion FN4   - Other, namely |
| 1. Wat is your age?  - Under 20 year - 20-24 year - 25-30 year - Over 30 year - I will not say |
| 1. What is your previous education?    - HAVO    - VWO    - MBO    - Inservice education    - Other, namely |
| **Thanks for sharing this background information!**  I will keep this confidential.  Now let's move on to the interview. |

| Interview | |
| --- | --- |
| **Start recorder** | Turn on audio recorder |
| *All variables, topics en central questions must be addressed* | |
| **Variables** and topics | **Central questions** and sub-questions |
| **Spiritual care**  Topic:   - View on spirituality | **In your own words, what means 'spiritual care' to you?**  Optional sub-question:   - Do you have an example of spiritual care? |
| **Learning process spiritual care competencies**  Topics:   - Influence curriculum - Influence EPICC Tool - Influence tutor - Influence internship - Influence Viaa - Other influences | **What are your experiences in learning to provide 'spiritual care'?**  Optional sub-questions :   - Do you have an example? - What influences your learning? - What helps you learn competence in spiritual care? - What doesn't help you learn competence in spiritual care? - What role does the teacher play? - What role does internship play? |
| **Experiences with EPICC Tool**  Topics:   - Selfscore - Using Experience - Structure EPICC Tool - Scoring competency - Open questions - Language | **What are your experiences in using the EPICC Tool?**  Optional sub- questions:   - How do you feel about evaluating/assessing yourself? - How did you experience the explanation of the questions? - What do you think of the structure of the questionnaire? - What do you think of the open questions? - What do you think of the use of language within the EPICC Tool? |
| **Influences on self-assessment**  Topics:   - Situation - Positive influence - Impeding influence | **Has anything (a situation or event) influenced your self-assessment on ‘spiritual care’ and what would you like to share about it?**  Optional sub-questions:   - What do you want to tell about this situation? - Do you have an example of this influence? - Is the influence positive as far as you are concerned or hindering? Why? |
| **Imbedding in education**  Topics:   - Moment of assessing (LZ, IZ, FN2, Stage FN3, FN3, FN4) - Link with assignments - Embedding in PPD - Frequency - Suggestions | **What is your opinion about the place within education, where the EPICC Tool is used?**  Optional sub-questions:   - What do you think of the EPICC Tool within the PPD- line? - Do you have any suggestions on what would be a good place for the EPICC Tool? - What do you think of the assignments to which the EPICC Tool is linked within PPD/internship? - What do you think about the frequency of collection? |
| **Suggestions for teaching competences spiritual care (control question)**  Topics:   - Tops - Tips | **Do you have suggestions for the programme to help students learn competences related to ‘spiritual care’?**  Optional sub-questions:   - What does the programme do well at the moment with regard to spiritual care? What tops do you give the programme in this respect? What should stay that way? - What would you like to see changed? - Do you have any tips for using the EPICC Tool? |
| **Suggestions for learning process spiritual care (control question)**  Topics:   - Suggestions | **If you had to give students some advice on how to increase/grow in their spiritual care competencies, what would it be?**  Optional sub-question:   - Do you have a concrete example? |

| Closing | |
| --- | --- |
| **Closing interview** | We come to the end of this interview.  **Is there anything not covered that you think is important to tell?**  **Is there anything else you would like to say?**  If you have issues of spirituality after the interview and you want to address them, I will refer you to studentbegeleiding@viaa.nl or your PPD- tutor. |
| **Thank you for the interview** | I would like to thank you for this interview!  Thank you also for your time!  **How did you find the interview?**  I hope you feel, that there was room for your story and that you were listened to. |
| **Leaving a phone number in case there are any questions** | If you have any further questions, you can contact me, Joanne Lassche-Scheffer. You can contact me by mail [j.lassche@viaa.nl](mailto:j.lassche@viaa.nl) or phone number: 038 202 4592 or via Teams |
