# Supplementary material for: Weaving spiritual care into nursing education: how the use of a self-assessment tool motivates students in a qualitative study
Source: BMC Nurs. 2026 Mar 4;25:346. doi: 10.1186/s12912-026-04514-1 (PMC13069700; doi:10.1186/s12912-026-04514-1)

## **Additional file 2. Drawings used during analysing process**

Content

[Additional file 2. Drawings used during analysing process 1](#_Toc222147556)

[*Figure1 Drawing 1* 2](#_Toc222147557)

[*Figure 2 Drawing 2* 3](#_Toc222147558)

[*Figure 3 Drawing 3* 4](#_Toc222147559)

[*Figure 4 Drawing 4* 5](#_Toc222147560)

[*Figure 5 Drawing 5* 5](#_Toc222147561)

[*Figure 6 Drawing 6* 6](#_Toc222147562)

Several drawings used to interpret and combine codes into themes in step 3 - 5 of Reflexive Thematic Analysis

### Figure1 Drawing 1


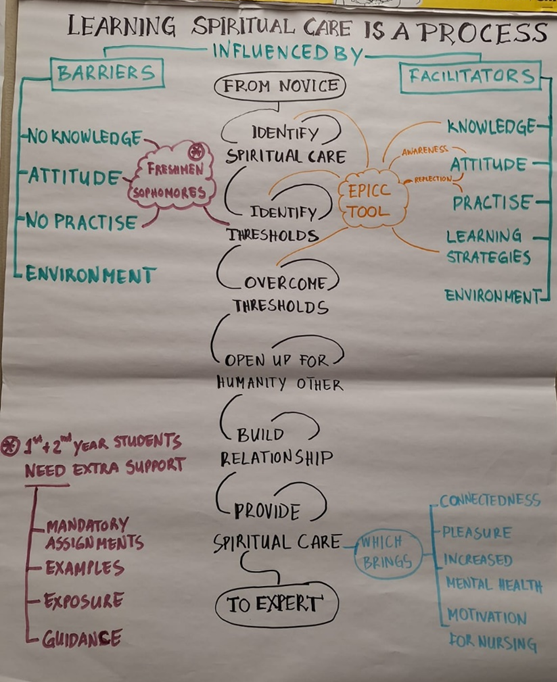


### Figure 2 Drawing 2


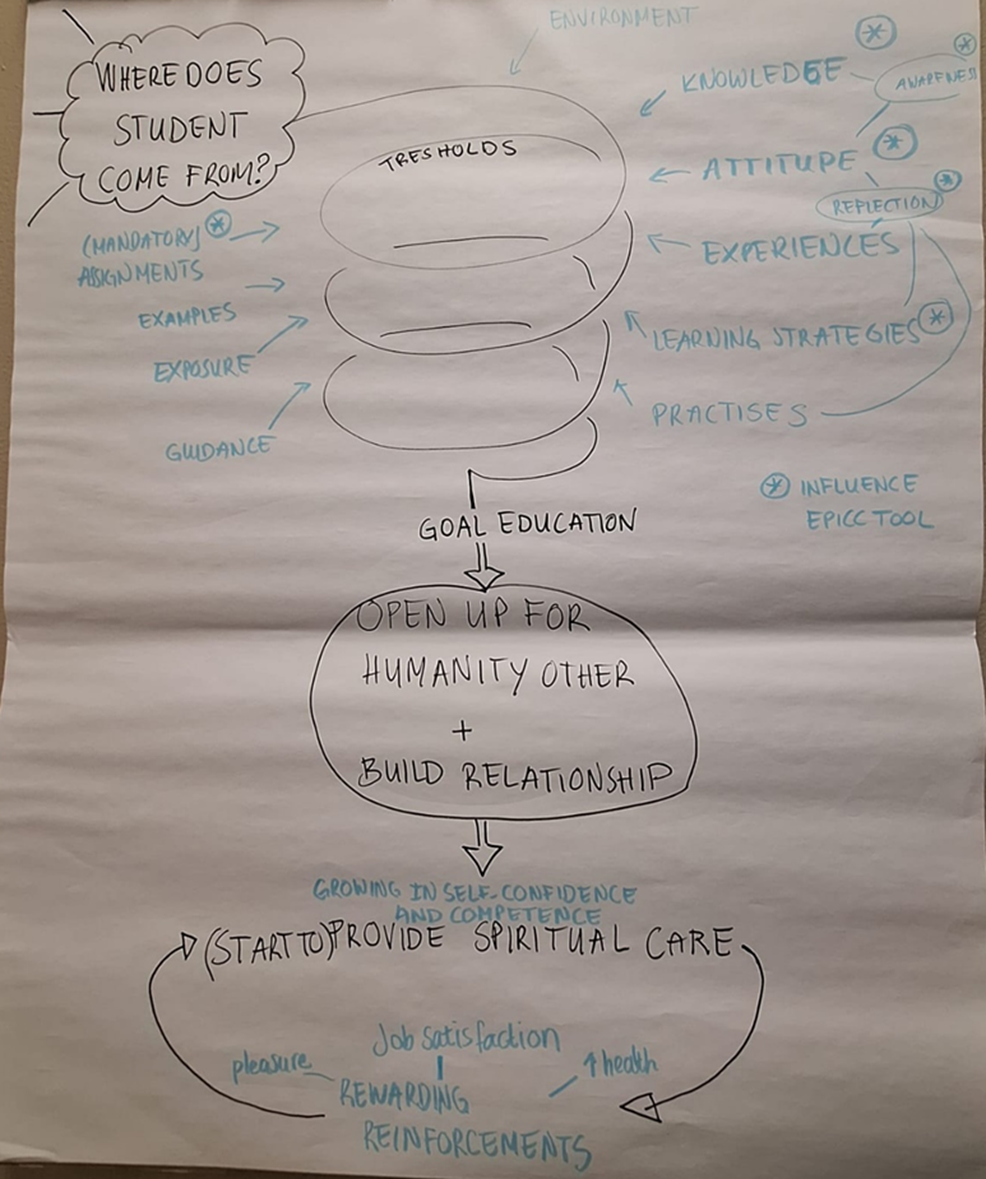


### Figure 3 Drawing 3


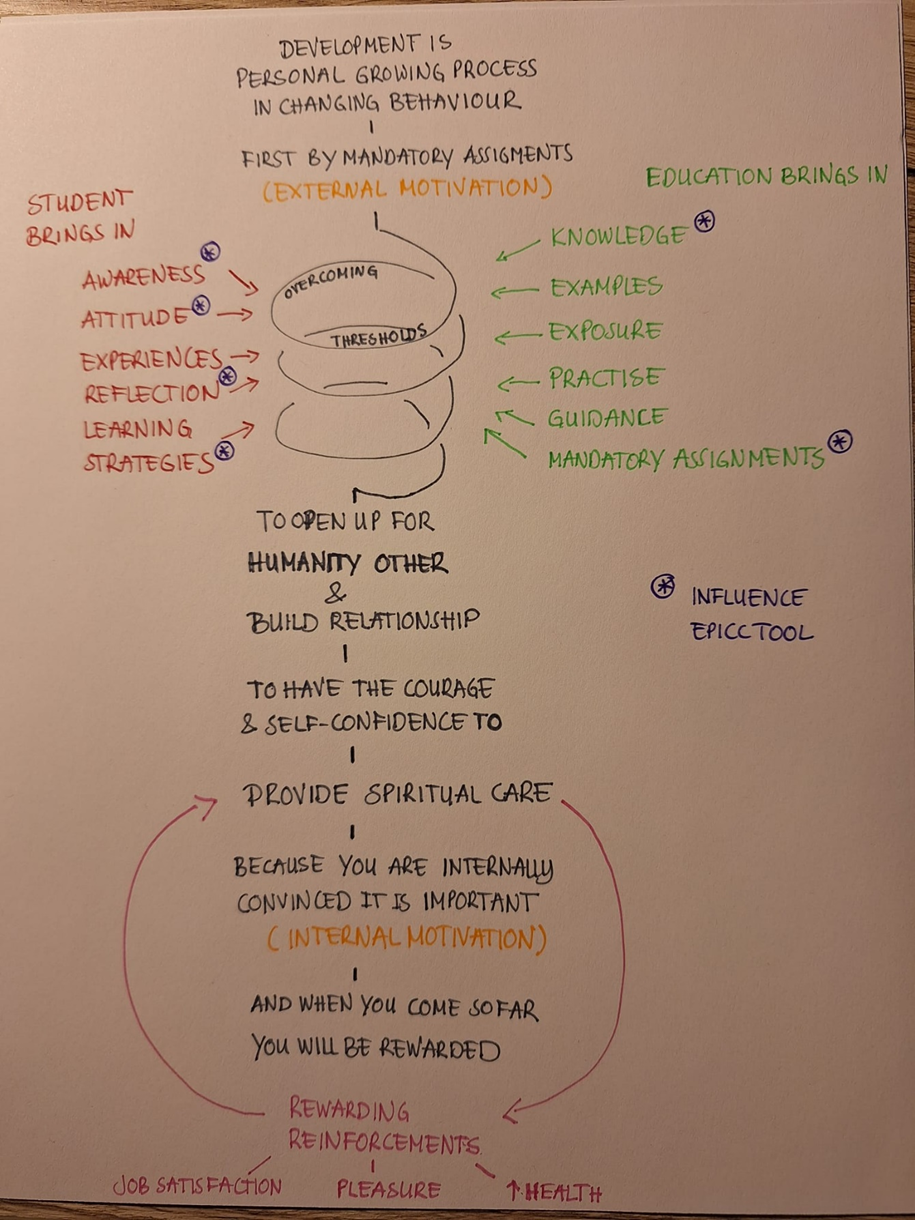


### Figure 4 Drawing 4

*
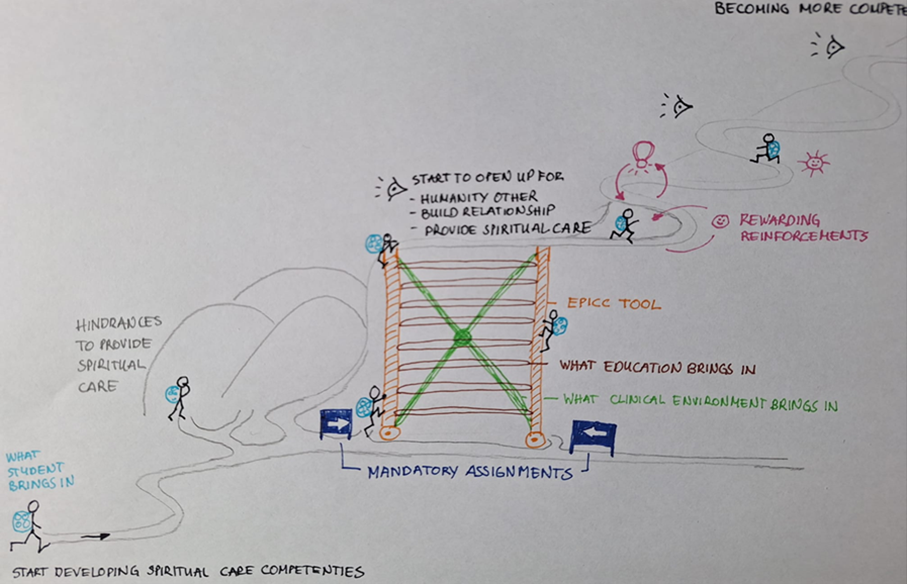
*

### Figure 5 Drawing 5


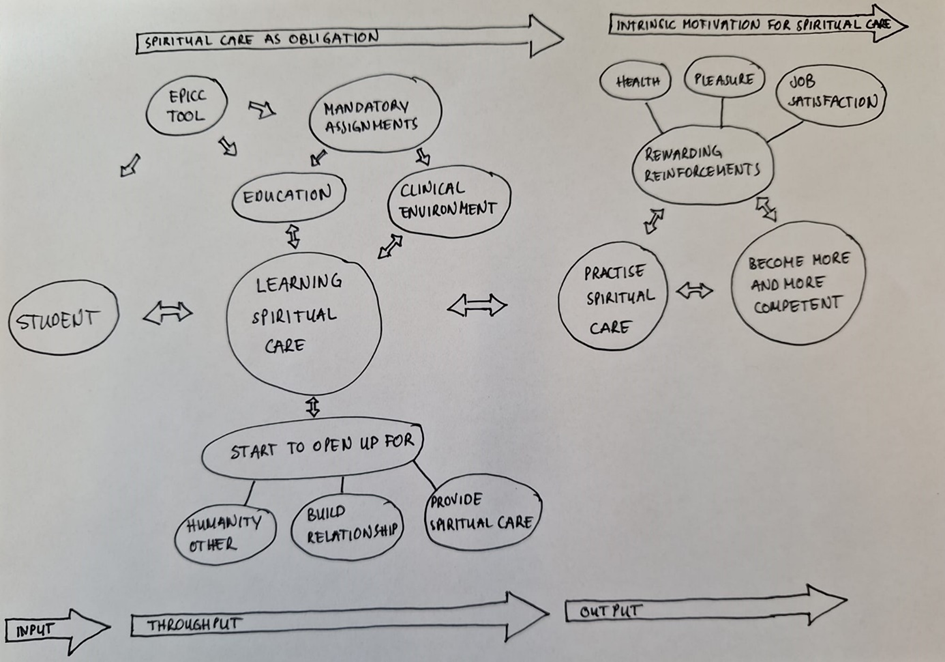


### Figure 6 Drawing 6


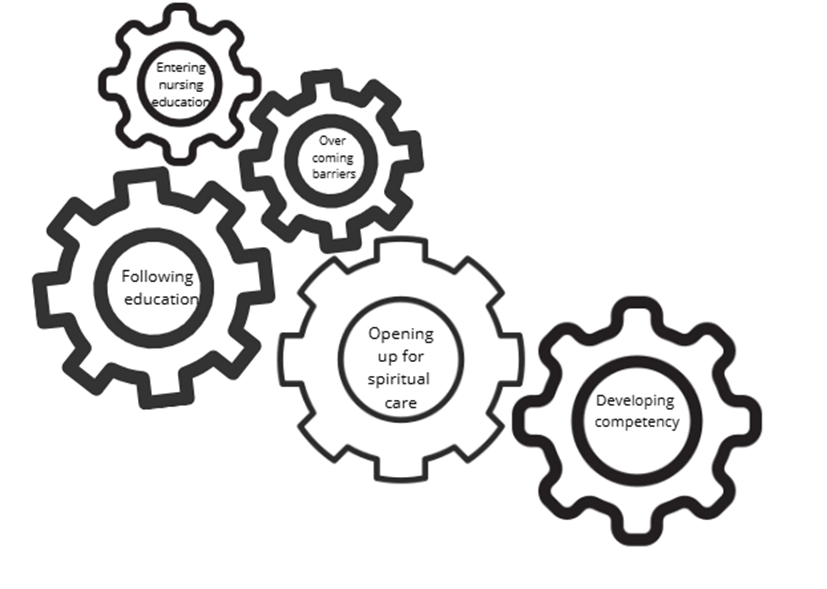

Supplement: Supplementary file 2 — Supplementary Material 2 [file 12912_2026_4514_MOESM2_ESM.docx]
